# Association between occupational exposure to mineral oil and rheumatoid arthritis: results from the Swedish EIRA case–control study

**DOI:** 10.1186/ar1824

**Published:** 2005-09-23

**Authors:** Berit Sverdrup, Henrik Källberg, Camilla Bengtsson, Ingvar Lundberg, Leonid Padyukov, Lars Alfredsson, Lars Klareskog, the Epidemiological Investigation of Rheumatoid Arthritis study group

**Affiliations:** 1 Rheumatology Unit, Department of Medicine, Karolinska Institutet/Karolinska Hospital, Stockholm, Sweden; 2 Rheumatology Unit, Eskilstuna Hospital, Eskilstuna, Sweden; 3 Institute of Environmental Medicine, Karolinska Institutet, Stockholm, Sweden; 4 Department of Occupational Medicine, National Institute for Working Life, Stockholm, Sweden; 5 Stockholm Center for Public Health, Stockholm County Council, Stockholm, Sweden

## Abstract

The aim of the present study was to investigate the association between exposure to mineral oil and the risk of developing rheumatoid arthritis (RA), and in addition to perform a separate analysis on the major subphenotypes for the disease; namely, rheumatoid factor (RF)-positive RA, RF-negative RA, anticitrulline-positive RA and anticitrulline-negative RA, respectively. A population-based case–control study of incident cases of RA was performed among the population aged 18–70 years in a defined area of Sweden during May 1996–December 2003. A case was defined as an individual from the study base who for the first time received a diagnosis of RA according to the American College of Rheumatology criteria of 1987. Controls were randomly selected from the study base with consideration taken for age, gender and residential area. Cases (*n *= 1,419) and controls (*n *= 1,674) answered an extensive questionnaire regarding lifestyle factors and occupational exposures, including different types of mineral oils. Sera from cases and controls were investigated for RF and anticitrulline antibodies.

Among men, exposure to any mineral oil was associated with a 30% increased relative risk of developing RA (relative risk = 1.3, 95% confidence interval = 1.0–1.7). When cases were subdivided into RF-positive RA and RF-negative RA, an increased risk was only observed for RF-positive RA (relative risk = 1.4, 95% confidence interval 1.0–2.0). When RA cases were subdivided according to the presence of anticitrulline antibodies, an increased risk associated with exposure to any mineral oil was observed only for anticitrulline-positive RA (relative risk = 1.6, 95% confidence interval = 1.1–2.2). Analysis of the interaction between oil exposure and the presence of HLA-DR shared epitope genes regarding the incidence of RA indicated that the increased risk associated with exposure to mineral oil was not related to the presence of shared epitope genotypes.

In conclusion, our study shows that exposure to mineral oil is associated with an increased risk to develop RF-positive RA and anticitrulline-positive RA, respectively. The findings are of particular interest since the same mineral oils can induce polyarthritis in rats.

## Introduction

Rheumatoid arthritis (RA) is a disease that is dependent on genetic factors as well as environmental factors, as seen from both concordance data in twins and from a number of epidemiological and genetic studies [1,2]. Whereas knowledge on the genetic basis of this disease is rapidly advancing [3,4], there is a scarcity of data on environmental agents that may cause arthritis [5-7]. In particular, very little information exists in humans on environmental risk factors with a capacity to induce arthritis in experimental arthritis systems.

Agents that are able to induce experimental arthritis in animals, particularly rodents, include a number of adjuvants originating from many different sources, including bacteria, yeast, viruses and mineral oils. Several models thus exist where rodents with certain genetic backgrounds develop arthritis after being exposed to nonimmunogenic adjuvants intracutaneously [8,9] or even percutaneously [10,11]. The exact mechanisms involved in the pathogenesis of these adjuvant arthritis models are still not completely understood, but we know that the adjuvants/mineral oils can activate cells within the lymph nodes without causing any simultaneous apparent inflammatory reaction in the skin [10]. Whether similar mechanisms (i.e. polyarthritis induced by simple adjuvants) are also operative in human arthritis is an open issue, although case reports exist on the occurrence of arthritis after immunization with Bacillus Calmette–Guerin [12,13], which is known to contain adjuvants able to cause arthritis in rodents [14].

In order to investigate the possible relationship between the occurrence of RA and the exposure to a series of different environmental agents, including simple adjuvants, we are currently performing a large population-based case–control study in Sweden using incident cases of RA. In the present report, we investigate the association between exposure to various mineral oils and the risk of developing RA.

## Materials and methods

The present study is a population-based case–control study of incident cases of RA among the population aged 18–70 years living in a geographically defined area in the middle part and southern part of Sweden during the period May 1996–December 2003. Ethical permission was obtained from relevant ethical committees and all the participants (cases as well as controls) consented to contribute to the study.

### Case identification

A case was defined as a person in the study base who for the first time received a diagnosis of RA according to the American College of Rheumatology criteria of 1987 [15]. As described previously [16], all potential cases were examined and diagnosed by a rheumatologist at the unit entering the case into the study. All rheumatology units linked to the general welfare system in the study area participated in the study, as well as almost all of the, very few, privately-run rheumatology units. In total there were 19 reporting clinics, 15 of which were 'Early Arthritis Clinics' [17]. At the start some centres also reported cases that did not satisfy the criteria in order to enable investigations of undifferentiated arthritis, but these subjects were eventually excluded from the study.

Analysis of rheumatoid factor (RF) was performed locally and reported as RF-positivity or RF-negativity. The RF levels were determined and the cutoff value was set to 20.

### Selection of controls

For each potential case a control was randomly selected from the study base with consideration taken for age, gender and residential area. The selection of controls was conducted using the national population register, which is continuously updated. If a control declined to participate, was not traceable or reported having RA, a new control was selected using the same principles (see also [16]). Controls belonging to cases excluded due to not fulfilling the American College of Rheumatology criteria remained in the study.

### Data collection

Information about environmental exposures was collected by an identical questionnaire given to the cases shortly after they had been informed about the RA diagnosis and sent by mail to the controls. All questionnaires were supposed to be answered at home.

Unanswered or incompletely answered questionnaires were completed by mail or by telephone by purpose-trained persons not connected to the rheumatology clinics. This was carried out in an identical way for the case and control groups. In total, 1,480 cases and 2,038 controls were identified. Of these, 1,419 cases (1,012 women and 407 men) and 1,674 controls (1,188 women and 486 men) participated in the study, giving a participation rate of 96% for cases and a participation rate of 82% for controls.

### Exposure

The questionnaire contains questions within a wide spectrum regarding personal circumstances, including lifestyle factors, occupational exposures, health aspects, socioeconomic factors and demographic data. Specific questions were asked about occupational exposure to cutting oil, motor oil, form oil, hydraulic oil and asphalt, respectively. This enabled the classification of cases and controls with regard to ever having been occupationally exposed to each of these mineral oils, respectively. Subjects who reported exposure to any of these mineral oils were classified as exposed to any mineral oil.

For each case the time point at which symptoms giving suspicion of RA started was used as an estimation of the disease onset. The year in which this time point occurred was defined as the index year. The same index year was used for the corresponding control. Only data on exposures up to the index year have been analysed in the present study.

### Detection of antibodies to citrulline-containing peptides

Antibodies to citrulline-containing peptides (anti-CP) were analysed with the Immunoscan-RA Mark2 ELISA test (see [18]). A level above 25 U/ml was regarded as positive according to instructions in the kit and validation at the clinical immunology laboratory in Uppsala. The kit uses cyclic citrullinated peptides as the substrate, and sera reacting positively with this kit are, in the present paper, defined as having significant antibody titres against citrullinated peptides (anti-CP^+^)

### Genotyping

DNA from ethylenediaminetetraacetic acid blood was extracted using the sequence-specific primer PCR method [19]. Among the HLA-DRB1 genes, DRB1*01, DRB1*04 and DRB1*10 were defined as 'shared epitope (SE) genes' [3,4]. At the beginning of the study, individuals from part of the material (81 cases) were subtyped for identification of HLA-DRB1*01 and HLA-DRB1*04 alleles. We determined 89% frequency of the DRB1*0101 allele and 98% frequency of the DRB1*0401 + DRB1*0404 + DRB1*0405 + DRB1*0408 alleles. For practical reasons the genotyping was restricted to only DR low-resolution analysis.

### Potential confounding factors

All results were adjusted for age and residential area according to the principle of control selection. In the analyses, age was categorised into 10 strata (18–24, 25–29, 30–34, 35–39, 40–44, 45–49, 50–54, 55–59, 60–64 and 65–70 years of age). Smoking and occupational class could also be considered as potential confounding factors. Smoking was categorised into two strata (never smokers and ever smokers) and occupational class was categorised into seven strata (unskilled manual workers, skilled manual workers, assistant non-manual employees, intermediate non-manual employees, higher non-manual employees, self-employed and farmers).

### Statistical analysis

Subjects who had been exposed to different mineral oils were compared with subjects unexposed to any mineral oil with regard to the incidence of RF^+ ^RA, RF^- ^RA, anti-CP^+ ^RA, anti-CP^- ^RA and RA overall, respectively, by calculating the odds ratio with the 95% confidence interval (CI). We performed matched analyses as well as unmatched analyses of the data. Odds ratios were adjusted for potential confounding by means of conditional logistic regression in the matched analyses and by means of unconditional logistic regression in the unmatched analyses. We only present results from the unmatched analyses as these were in close agreement with those from the matched analyses but, in general, had higher precision. Odds ratios were interpreted as the relative risk (RR) because the study was population based. Results for women and men were analysed separately. Estimates of RR were adjusted for potential confounding from age, gender, residential area and smoking. Further adjustment for occupational class only marginally changed the estimates and was not retained in the final analyses.

The presence of HLA-DR SE genes is a risk factor for RF^+ ^RA and anti-CP^+ ^RA, but not for either RF^- ^RA or anti-CP^- ^RA [20,21]. It is thus of interest to investigate the possibility of a gene–environment interaction between SE genes and exposure to mineral oil regarding the incidence of RF^+ ^RA and anti-CP^+ ^RA, respectively. Interaction between the genotype and mineral oil was evaluated using departure from the additivity of effects as a criterion of interaction, as suggested by Rothman and colleagues [22]. To quantify the amount of interaction, the attributable proportion due to interaction was calculated together with the 95% CI [23]. The attributable proportion due to interaction, which takes a value between 0 and 1, is the proportion of the incidence among persons exposed to two interacting factors that is attributable to the interaction *per se *(i.e. reflecting their joint effect beyond the sum of their independent effects). A potential interaction between smoking and mineral oil was also evaluated. All analyses were performed using the Statistical Analysis System (version 8.2; SAS Institute, Stockholm, Sweden).

## Results

Of a total of 1,419 cases in this study, 1,012 were women and 407 were men (mean age at inclusion of 50 and 53 years, respectively). A total 65.5% of the female cases and 66.3% of the male cases were RF^+^. The mean duration of disease at inclusion in the study was 10 months. Only men reported a substantial occupational exposure to mineral oils (in total 135 cases and 132 controls), with occupational exposure to mineral oils uncommon among women (21 cases and 21 controls). Only men were retained in the further analysis. Among these men, motor oil (84 cases and 84 controls) and hydraulic oil (83 cases and 72 controls) were the most common exposures.

Exposure to any mineral oil was associated with a 30% increased risk of developing RA (RR = 1.3, 95% CI = 1.0–1.7) (Table 1). When cases were subdivided into RF^+ ^RA and RF^- ^RA, an increased risk was only observed for RF^+ ^RA (RR = 1.4, 95% CI = 1.0–2.0). The same pattern with a higher RR associated with RF^+ ^RA was observed for all of the specific mineral oils. The RR of developing RF^+ ^RA associated with exposure to hydraulic oil was 1.5 (95% CI = 1.0–2.3).

We also investigated the relationship between exposure to different oils and the incidence of anti-CP^+ ^RA and anti-CP^- ^RA, respectively (Table 2). Exposure to any mineral oil was associated with a 60% increased risk of anti-CP^+ ^RA (RR = 1.6, 95% CI = 1.1–2.2). Higher RR associated with anti-CP^+ ^RA as compared with anti-CP^- ^RA was seen for all the specific mineral oils. The RR of developing anti-CP^+ ^RA associated with exposure to hydraulic oil was 1.7 (95% CI = 1.1–2.6), and that for motor oil was 1.5 (95% CI = 1.0–2.3).

In the analysis, adjustment was made according to age, residential area and smoking. The results after adjustment for smoking were almost identical to those not adjusted for smoking (Tables 1 and 2).

**Figure 1:**
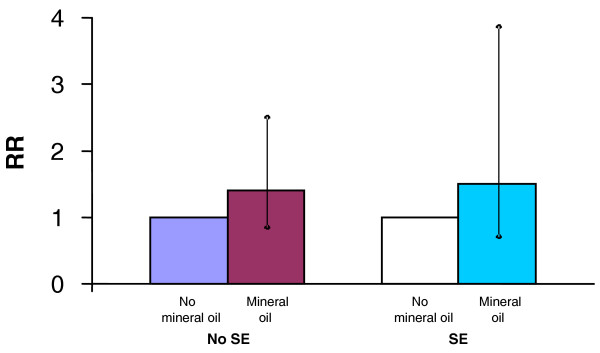
Relative risk of anti-CP-positive RA with mineral exposure and different expression of SE genes. The relative risk (RR) of developing rheumatoid arthritis (RA) positive for antibodies to citrulline-containing peptides (anti-CP) with mineral exposure and shared epitope (SE) genes compared with unexposed subjects with SE genes is 1.4, with 95% confidence interval = 0.8–2.4. The corresponding risk among subjects with no SE gene is 1.5, with 95% confidence interval = 0.6–3.9.

The presence of HLA-DR SE genes is a risk factor for RF^+ ^RA and anti-CP^+ ^RA, but not for either RF^- ^RA or anti-CP^- ^RA [20,21]. When we analysed the possibility of a gene–environment interaction between SE genes and exposure to mineral oil regarding the incidence of RF^+ ^RA and anti-CP^+ ^RA, respectively, no strong evidence of such an interaction was found (Table 3 and Fig. 1). The attributable proportion due to the interaction between SE genes and mineral oil was 0.2 (95% CI = -0.2-0.6) regarding RF^+ ^RA as well as regarding anti-CP^+ ^RA.

When a potential interaction between smoking and mineral oils was analysed, a tendency towards such an interaction was noted but a firm conclusion was hampered by the small numbers (regarding anti-CP^+ ^RA, the attributable proportion due to interaction was 0.5 [95% CI = -0.2-1.2]).

## Discussion

According to the results of this study, males exposed to various mineral oils in their profession appear to have an increased risk of developing RA. This observation is of interest in the search for aetiological factors of importance for triggering RA, as exposure to the same kinds of oils have been shown to induce polyarthritis with large similarities to RA in rodents [8,9].

This study has the advantage of being a population-based case–control study using only incident cases of new diagnosed RA, fulfilling the American College of Rheumatology criteria, assessed by a specialist in rheumatology. All rheumatology units linked to the general welfare system in the study area reported cases to the study, as did privately-run rheumatology units [17]. Cases received their questionnaire in connection with inclusion into the study at each study centre (i.e. in connection with the time point of the RA diagnosis), whereas the controls received their questionnaires by mail. All questionnaires were supposed to be answered at home. Both cases and controls returned their questionnaire by mail to the study secretariat at the Karolinska Institutet. It is unlikely that the differential distribution process of the questionnaire to cases and to controls, respectively, led to any important difference regarding the quality of exposure information.

A possible disadvantage with a case–control study with retrospective collection of exposure data is the risk for misclassification of exposure due to a recall bias that differs between cases and controls. Only subjects that received a diagnosis of RA for the first time were included in order to reduce the risk for recall bias, the mean duration between the estimated disease onset and inclusion into the study was 10 months, and analyses of data of environmental exposures were only performed up to the index year. Bias due to change in habits, job or work exposure as a result of the disease was therefore probably limited. In order to investigate whether misclassification of exposure to mineral oils differed between cases and controls, an industrial hygienist performed an independent classification based on information regarding occupation and the branch of industry during the period of the stated exposure to mineral oils. The industrial hygienist was blinded with regard to the disease status of the individuals. According to the result of this investigation a similar proportion (15% and 20%) of cases and controls seemed to be false positive with regard to exposure to mineral oils. The assessment made by the hygienist should not be regarded as more qualified than the assessment made by the subjects. However, the marked and similar correspondence of the hygienist's assessment and the subjects' statements among cases and controls, respectively, suggests that differential misclassification of exposure has probably not biased the estimated RR to any great extent. The response rate in the study was high, with 96% for cases and 82% for controls, which limits risk for selection bias in this stage.

All results were adjusted for age and residential area according to the principle of control selection. In the analysis, we investigated the potential confounding from smoking. Adjustment for smoking only marginally changed the estimated RR (Tables 1 and 2). Hence, differences regarding smoking habits do not explain the observed association between exposure to mineral oils and the risk of RA. When a potential interaction between smoking and mineral oils was analysed, a tendency towards such an interaction was noted, but the observation was based on a small number.

According to the results of our study, mineral oils (in particular, hydraulic oil and motor oil) appear to be associated with a particular high risk of RF^+ ^RA and anti-CP^+ ^RA. Bearing in mind the relatively small number of exposed men, however, caution is warranted regarding any far-reaching conclusion about particular mineral oils.

Previously, to our knowledge, only one study has investigated the relationship between exposure to mineral oils and the development of RA [24]. This is notable, since mineral oils have been very well documented as arthritogenic agents in rodents [8]. The administration of mineral oils, therefore, both intracutaneously as one single injection and percutaneously in multiple exposures, has been shown to induce an erosive and RA-like polyarthritis in certain strains of rats, in particular the DA rat strain [10,11].

Our results confirm those of previous studies that the presence of HLA-DR SE genes is a risk factor for anti-CP^+ ^RA and RF^+ ^RA but not for either anti-CP^- ^RA or RF^- ^RA [20,21]. Analysis of a possible interaction between the SE genes and exposure to mineral oils did not reveal any significant interaction between exposure to mineral oil and the presence of HLA-DR SE genes. Although based on small numbers of observations, this suggests that mechanisms responsible for the association between mineral oil exposure and RA may be different from those responsible for the association between smoking and RA, where a pronounced interaction between smoking and the HLA-DR SE genes was observed [20,21].

The potential molecular pathogenesis of polyarthritis associated with exposure to mineral oil is difficult to speculate on. It is known in experimental animals, however, that oils and other adjuvants confer their activation of the immune system mainly in the lymph nodes, without leaving any signs of inflammation in the exposed skin [11,25-28]. Here, an initial activation of the innate immune system subsequently leads to the activation also of T-cell immunity, in such a way that the T cells can subsequently transfer the disease to naive animals [29]. It is also known in rodents that susceptibility to adjuvant-induced arthritis, including oil-induced disease, is highly dependent on the genetic constitution of the exposed animals [26]. It is thus possible that humans with a genetic constitution similar to the adjuvant-susceptible rodents would also have a particularly high susceptibility for mineral-oil associated arthritis. This question may soon be possible to investigate further if precise polymorphic genes associated with susceptibility to adjuvant arthritis in rodents are identified, and indications that such genes exist have already been provided [30].

It is thus possible to hypothesise that adjuvant stimulus of the innate immune system, taking place in genetically susceptible human beings, would trigger the activation of a cascade of events involving activated T lymphocytes, and that these events for as yet unknown reasons finally result in inflammatory joint disease. In this context it is of interest that the association between RA and exposure to mineral oil does not appear to be related to the presence or absence of HLA-DR SE genes in the mineral-exposed individuals. In conjunction with another environmental exposure – smoking – a major interaction was seen between smoking and the presence of HLA-DR SE. This interaction has been suggested to depend on the capacity of smoke to induce an aberrant citrullination of proteins in the lungs of long-term smokers, something that may trigger anti-CP immunity in individuals carrying HLA-DR SE genes [21].

The present demonstration of an exposure not linked to the presence of HLA-DR SE genes indicates a complex pattern of interactions between several environmental triggers, several genetic features and, eventually, several patterns of immunoreactivities in the pathogenesis of RA. If these different patterns in humans can be linked to different rodent models of arthritis – as is suggested from the findings in the present paper – we may be able to use knowledge of molecular pathogenesis and targeted therapies gained in different animal systems to develop a better understanding of both pathogenesis and treatment of relevant subgroups of the human disease.

## Conclusion

The present study shows that exposure to an environmental agent capable of inducing an RA-like polyarthritis in rodents – mineral oil – is associated with an increased risk for RF^+ ^RA and anti-CP^+ ^RA in man. Further exploration of this finding may be of interest in elucidating whether other types of adjuvants, such as microbial agents and other occupational agents, can also act as arthritis-inducing agents in humans, and to further link the molecular pathogenesis of adjuvant-associated arthritis in rodents with adjuvant-induced arthritis in man.

## Abbreviations

anti-CP = antibodies to citrulline-containing peptides; CI = confidence interval; EIRA = Epidemiological Investigation of Rheumatoid Arthritis; ELISA = enzyme-linked immunosorbent assay; PCR = polymerase chain reaction; RA = rheumatoid arthritis; RF = rheumatoid factor; RR = relative risk; SE = shared epitope.

## Competing interests

The author(s) declare that they have no competing interests.

## Authors' contributions

BS contributed to the design of the study, and the interpretation and writing of the manuscript. HK performed a major part of the biostatistics work and contributed to the interpretation of results and the writing of the manuscript. CB contributed to the design of the study, to the collection of the data, to statistical analysis and to the writing of the manuscript. IL contributed to the design of the study and to the interpretation of the results. LP had the main responsibility for the genetic analyses and contributed to the analysis and interpretation of the results. LA and LK were responsible for the overall design of the Epidemiological Investigation of Rheumatoid Arthritis study, for the analysis of data and for the final writing of the manuscript. All authors read and approved the final text before submission of the manuscript.

## Figures and Tables

**Table 1:** Relative risk for developing RF-positive or RF-negative RA in men exposed to mineral oils

Outcome	Oil	Exposed cases/controls (*n*)^a^	Relative risk^b^	95% confidence interval	Relative risk^c^	95% confidence interval
Total RA	Cutting fluid	36/39	1.1	0.7–1.9	1.1	0.7–1.8
	Motor oil	84/84	1.2	0.9–1.8	1.2	0.9–1.8
	Hydraulic oil	83/72	1.4	1.0–2.1	1.4	1.0–2.0
	Form oil	25/24	1.3	0.7–2.5	1.3	0.7–2.4
	Asphalt	13/14	1.3	0.6–2.8	1.2	0.6–2.7
	Any mineral oil	135/132	1.3	1.0–1.7	1.3	1.0–1.7
RF^+ ^RA	Cutting fluid	29/39	1.4	0.8–2.5	1.4	0.8–2.4
	Motor oil	60/84	1.4	0.9–2.1	1.4	0.9–2.1
	Hydraulic oil	56/72	1.6	1.0–2.3	1.5	1.0–2.3
	Form oil	20/24	1.7	0.9–3.3	1.7	0.9–3.2
	Asphalt	9/14	1.4	0.6–3.5	1.4	0.6–3.4
	Any mineral oil	96/132	1.5	1.0–2.0	1.4	1.0–2.0
RF^- ^RA	Cutting fluid	7/39	0.6	0.2–1.4	0.6	0.2–1.4
	Motor oil	24/84	0.9	0.6–1.6	0.9	0.5–1.6
	Hydraulic oil	27/72	1.2	0.7–2.1	1.2	0.7–2.1
	Form oil	5/24	0.6	0.2–1.8	0.6	0.2–1.8
	Asphalt	4/14	0.9	0.3–3.0	0.9	0.3–3.0
	Any mineral oil	39/132	1.0	0.6–1.5	1.0	0.6–1.5

Relative risk and 95% confidence interval for developing rheumatoid factor (RF)-positive rheumatoid arthritis (RA), RF^- ^RA and RA overall (total RA) for men 18–70 years old exposed to different kinds of mineral oils compared with unexposed men.

^a^RF status unknown for one unexposed case.

^b^Adjusted for age and residential area.

^c^Adjusted for age, residential area and smoking.

**Table 2:** Relative risk for developing anti-CP-positive RA or anti-CP-negative RA for men exposed to mineral oils

Outcome	Oil	Exposed cases/controls (*n*)	Relative risk^a^	95% confidence interval	Relative risk^b^	95% confidence interval
Anti-CP^+ ^RA	Cutting fluid	28/39	1.6	0.9–2.7	1.5	0.8–2.6
	Motor oil	57/84	1.5	1.0–2.2	1.5	1.0–2.3
	Hydraulic oil	56/72	1.8	1.2–2.7	1.7	1.1–2.6
	Form oil	16/24	1.5	0.7–2.9	1.4	0.7–2.9
	Asphalt	10/14	1.6	0.7–3.9	1.5	0.6–3.7
	Any mineral oil	93/132	1.6	1.1–2.2	1.6	1.1–2.2
Anti-CP^- ^RA	Cutting fluid	8/39	0.6	0.2–1.3	0.6	0.2–1.3
	Motor oil	27/84	0.9	0.6–1.5	0.9	0.6–1.5
	Hydraulic oil	27/72	1.0	0.6–1.7	1.0	0.6–1.7
	Form oil	9/24	1.1	0.5–2.6	1.1	0.5–2.6
	Asphalt	3/14	0.7	0.2–2.6	0.7	0.2–2.6
	Any mineral oil	42/132	1.0	0.6–1.5	1.0	0.6–1.5

Relative risk and 95% confidence interval for developing antibodies to citrulline-containing peptides (anti-CP)-positive rheumatoid arthritis (RA) and anti-CP^- ^RA for men 18–70 years old exposed to different kinds of mineral oils compared with unexposed men.

^a^Adjusted for age and residential area.

^b^Adjusted for age, residential area and smoking.

**Table 3:** Relative risk for RA with different combinations of mineral oil exposure and shared epitope genes

Outcome	Exposure to mineral oil	No shared epitope			Any shared epitope		
		
		Cases/controls (*n*)^a^	Relative risk^b^	95% confidence interval	Cases/controls (*n*)^a^	Relative risk^b^	95% confidence interval
RF^+ ^RA	No	28/89	1.0^c^		128/88	4.7	2.8–8.0
	Yes	17/43	1.2	0.6–2.5	73/38	6.4	3.4–11.8
RF^- ^RA	No	32/89	1.0^c^		63/88	2.1	1.2–3.6
	Yes	10/43	0.7	0.3–1.5	25/38	2.1	1.1–4.2
Total RA	No	60/89	1.0^c^		191/88	3.2	2.1–4.9
	Yes	27/43	0.9	0.5–1.7	98/38	3.9	2.3–6.6
							
Anti-CP^+ ^RA	No	18/89	1.0^c^		126/88	8.0	4.4–14.7
	Yes	13/43	1.6	0.7–3.7	75/38	11.1	5.6–22.1
Anti-CP^- ^RA	No	42/89	1.0^c^		65/88	1.5	0.9–2.5
	Yes	14/43	0.7	0.3–1.5	23/38	1.5	0.7–2.9
Total RA	No	60/89	1.0^c^		191/88	3.2	2.1–4.9
	Yes	27/43	0.9	0.5–1.7	98/38	3.9	2.3–6.6

Relative risk and 95% confidence interval for developing rheumatoid factor (RF)-positive rheumatoid arthritis (RA), RF^- ^RA, antibodies to citrulline-containing peptides (anti-CP)-positive RA, anti-CP^- ^RA and total RA for men with different combinations of exposure to mineral oil and shared epitope genes compared with men unexposed to any mineral oil and lacking shared epitope genes.

^a^Data on the shared epitope are missing for 10 cases and 51 controls exposed to mineral oil, and for 21 cases and 176 controls unexposed to mineral oil (in comparison with Tables 1 and 2).

^b^Relative risk adjusted for age, residential area and smoking.

^c^Reference group.
